# The application of deep learning for the classification of correct and incorrect SNP genotypes from whole-genome DNA sequencing pipelines

**DOI:** 10.1007/s13353-020-00586-0

**Published:** 2020-09-29

**Authors:** Krzysztof Kotlarz, Magda Mielczarek, Tomasz Suchocki, Bartosz Czech, Bernt Guldbrandtsen, Joanna Szyda

**Affiliations:** 1 Biostatistics Group, Department of Genetics, Wroclaw University of Environmental and Life Sciences, Kozuchowska 7, 51-631 Wroclaw, Poland; 2 Institute of Animal Breeding, Balice, Poland; 3 Animal Breeding Group, Department of Animal Sciences, University of Bonn, Bonn, Germany

**Keywords:** Classification, Keras, Next-generation sequencing, Python, SNP calling, SNP microarray, TensorFlow

## Abstract

A downside of next-generation sequencing technology is the high technical error rate. We built a tool, which uses array-based genotype information to classify next-generation sequencing–based SNPs into the correct and the incorrect calls. The deep learning algorithms were implemented via Keras. Several algorithms were tested: (i) the basic, naïve algorithm, (ii) the naïve algorithm modified by pre-imposing different weights on incorrect and correct SNP class in calculating the loss metric and (iii)–(v) the naïve algorithm modified by random re-sampling (with replacement) of the incorrect SNPs to match 30%/60%/100% of the number of correct SNPs. The training data set was composed of data from three bulls and consisted of 2,227,995 correct (97.94%) and 46,920 incorrect SNPs, while the validation data set consisted of data from one bull with 749,506 correct (98.05%) and 14,908 incorrect SNPs. The results showed that for a rare event classification problem, like incorrect SNP detection in NGS data, the most parsimonious naïve model and a model with the weighting of SNP classes provided the best results for the classification of the validation data set. Both classified 19% of truly incorrect SNPs as incorrect and 99% of truly correct SNPs as correct and resulted in the F1 score of 0.21 — the highest among the compared algorithms. We conclude the basic models were less adapted to the specificity of a training data set and thus resulted in better classification of the independent, validation data set, than the other tested models.

**Electronic supplementary material:**

The online version of this article (10.1007/s13353-020-00586-0) contains supplementary material, which is available to authorized users.

## Introduction

Next-generation sequencing (NGS) technology has led to a tremendous increase in sequencing speed and a decrease in sequencing cost. It allows fast and cost-effective sequencing of whole genomes of many individuals. The downside of sequencing carried out by high-throughput processes are the significant technical (Pfeiffer et al. 2018; Ma et al. 2019) and bioinformatics (Abnizova et al. 2017) error rates. In particular, the very large amounts of genomes sequenced with moderate or low coverage, short-read lengths and individual genetic variation often cause numerous computational problems (Horner et al. 2010). Such drawbacks make utilizing NGS data for research dependent on bioinformatics tools for data editing and processing. The resulting polymorphism detected should therefore rather be regarded as an estimate of the true underlying genetic variation. Therefore, it should be kept in mind that not all of the reported polymorphisms represent true variants. In addition, some true polymorphisms remain undetected despite the availability of the whole-genome sequence. A trivial example of such situations is differences in the number and positions of detected polymorphisms resulting from the application of various bioinformatics pipelines to the same set of short-read sequence data (e.g. Hwang et al. 2015; Laurie et al. 2016). Also, in our unpublished analyses of 197 cattle genomes, with an average genome coverage of 10×, we observed differences between single-nucleotide polymorphism (SNP) called using GATK (McKenna et al. 2010), FreeBayes (Garrison and Marth 2012) and SAMtools (Li et al. 2009). The differences amounted to some 25% depending on the particular chromosome.

Therefore, in the current study, we aimed to build a tool, which uses standard information available in the Variant Calling Format (VCF) file for the classification of SNPs into correct and incorrect detections. Based on the available data, which comprises individuals genotyped by a commercial high-density oligonucleotide microarray and with whole-genome sequence, we were able to identify correct and incorrect SNP detections. The rate of SNPs incorrectly called from NGS data—represented by polymorphisms with discordant genotypes between a microarray and NGS—was only 2%. Although it is a positive observation, it makes it difficult to use standard statistical tools for classification since, with such a severe imbalance in class counts, we enter a rare event data problem. For such data, a standard logistic regression-based classifier does not apply because of a bias in maximum likelihood-derived parameter and probability estimates, which is due to the strong imbalance between the observed counts of events and non-events (King and Zeng 2001a, b). Even though a modification of logistic regression based on bootstrap and Markov chain Monte Carlo (Frühwirth-Schnatter and Wagner 2008) dedicated to the rare event data was applied for the analysis of the data, parameter estimation and subsequent classification did not improve. Clearly, a valid possibility to improve the model would be to add more independent variables. In practice, the number of explanatory variables is limited by the information included in VCF files. On the other hand, machine learning algorithms, including deep learning (DL), proved to provide a flexible classification tool for diverse and complex data structures (Jiang et al. 2020). DL has been successfully implemented in many classification challenges naming, for example, the KAGGLE competition (https://www.kaggle.com). For livestock data, the application of DL has just begun. It has mainly focussed on the prediction of animals’ genetic merit (for a review, see Pérez-Enciso and Zingaretti 2019). The use of DL-based tools for NGS variant detection and/or classification is still scarce. It has only recently been considered by Ravasio et al. (2018), Singh and Bhatia (2019) and Gupta and Saini (2020). In the current study, we exploited the potential of DL to classify correct and incorrect SNP discoveries for the context of livestock whole-genome sequence data. Beginning from a naïve classification algorithm, we further moved towards its modifications aiming to mitigate the problem of the very low number of SNPs representing the incorrect SNP class. Moreover, we also explored the continuous [0, 1] space of the distribution of SNP class probability estimated by the deep learning network in order to determine the best cutoff for SNP binary classification. Since the study aims to provide a general classifier, we used explanatory variables available in a standard VCF file.

## Materials and methods

### Datasets

The data comprised whole-genome DNA sequence reads of four traditional Danish Red Dairy Cattle bulls. Samples were sequenced by the Illumina HiSeq2000 platform with paired-end 100 bp read length with a 300 bp insert size. The total number of raw reads generated for a single animal varied between 249,478,818 and 290,364,464. This resulted in the average genome coverage of 10x. Additionally, these bulls were genotyped using the Illumina BovineHD BeadArray comprising 777,962 SNPs. This data was then utilised to compose the following subsets:

- The training data set, used for building a DL-based classifier, was composed of three (out of the four) animals.
- The validation data set, used as the independent input for the validation of the classification quality, comprised data from the fourth animal with NGS SNPs identified based on the same pipeline as in the training data set.

### SNP calling

A pipeline for SNP calling comprised the alignment to the reference genome carried out by using BWA-MEM (Li and Durbin 2009) with the following default parameters: the seed length of 19, the matching score of 1, mismatch penalty of 4, gap open penalty of 6 and gap extension penalty of 1. Post-alignment processing included the conversion of SAM-formatted files into the binary (BAM) format, data indexing and marking of PCR duplicates. This step was performed using Picard (https://broadinstitute.github.io/picard/) and SAMtools. Pre-variant calling was implemented via the GATK package and included local re-alignment around INDELs by using GATK’s RealignerTargetCreator and IndelRealigner tools followed by quality score recalibration by using BaseRecalibrator and PrintReads tools. For the actual variant calling, the UnifiedGenotyper tool from the GATK package was used. Note that the above pipeline was run only for those 772,173 SNPs genomic positions, which were also genotyped by the Illumina BovineHD BeadArray and were defined in the SNPchiMp (Nicolazzi et al. 2015) database.

### Correct and incorrect SNP definition

After the exclusion of missing genotypes, the total number of the analysed polymorphic loci for each of the bulls was 764,446 and these were compared between the NGS and the microarray outputs. Correct SNPs refer to a full agreement in genotypes estimated by both technologies; incorrect SNPs were defined as mismatches involving at least one allele. For the case of multi-allelic SNPs, when two or more alternative alleles were called in the NGS output, we checked if at least one of these alleles allowed for a match with the genotype called by a microarray.

### Explanatory variables

The following explanatory variables from a standard VCF output were considered for the classification:

- The probability of incorrectly called alternative allele (QUAL): QUAL = − 10log_10_(*P*_1_), where *P*_1_ denotes the probability that the identified alternative allele is an incorrect detection.
- The conditional likelihood of incorrectly called alternative allele (GQ): GQ = − 10log_10_(*P*_2_), where *P*_2_ denotes the probability that the identified genotype is an incorrect detection conditional on the position being polymorphic.
- Sequencing depth at the polymorphic site combined over all four sequenced individuals (DP).
- Sequencing depth at the polymorphic site for a given individual (DP2).
- The coded genotype (CALL): $$ \mathrm{CALL}=\left\{\ \begin{array}{c}1\kern0.5em \mathrm{for}\kern0.5em ./.\\ {}\kern0.75em 2\kern0.5em \mathrm{for}\kern0.5em 0/0\\ {}\kern0.75em 3\kern0.5em \mathrm{for}\kern0.5em 0/1\\ {}\kern1em 4\kern0.5em \mathrm{for}\kern0.5em 1/1\ \end{array}\right. $$.
- A categorical variable was constructed based on three reference bases downstream of a SNP.
- A categorical variable was constructed based on three reference bases upstream of a SNP.

The last three variables were included into modelling in order to capture the potential sequencer errors specific to the fluorescence of particular genotypes (CALL) or the sequence closely neighbouring the polymorphic site.

### Deep learning algorithms

**Fig. 1:**
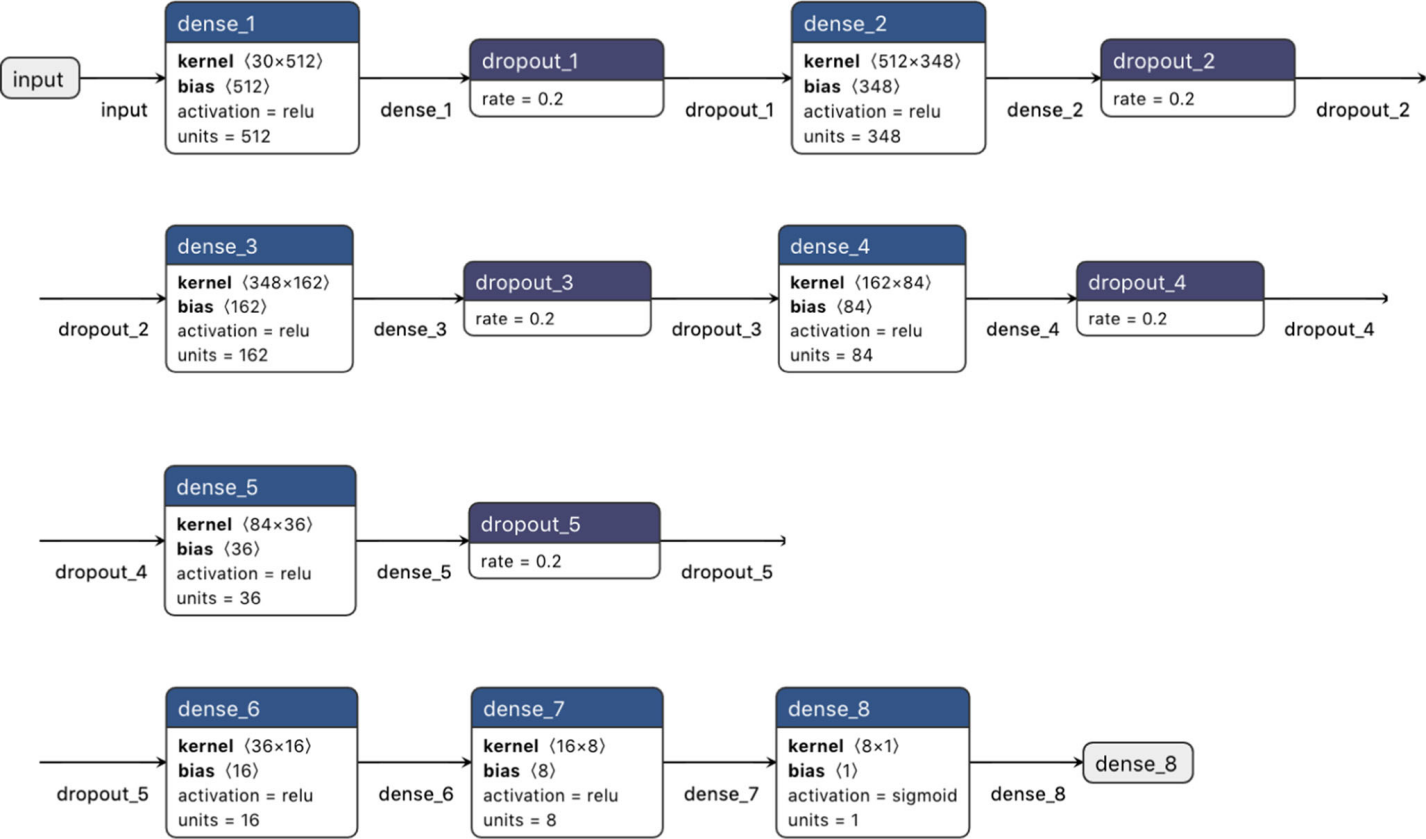
Implementation scheme for the NAÏVE deep learning algorithm for SNP classification in Keras

The deep learning algorithms were implemented via the Keras interface (Chollet 2015) with the TensorFlow (Abadi et al. 2015) library in Python 3.7.7 on a personal computer running Windows 10, with an Intel Core i5-3210M CPU 2.50GHz (2 cores and 3-MB cache memory), 8 GB RAM and 120 GB of SSD. Prior to the implementation of the algorithm, all quantitative explanatory variables (QUAL, GQ, DP and DP2) were transformed into the standard normal distribution. The categorical explanatory variables (base trio up- and downstream of a SNP as well as CALL) were one-hot encoded. The network architecture underlying the naïve algorithm (NAÏVE) was composed of eight sequentially connected layers with gradually decreasing numbers of parameters and a dropout rate of 0.2 after the first five layers. The dropout algorithm modifies the activation matrix (***X***) estimated by every single network by randomly setting 20% of its values to zero. In the first seven layers, the rectified linear unit (*ReLU*) function, given by *f*(*x*) = max(0, *x*_*ij*_), was used for activation, while the *sigmoid*, given by $f(x)=\frac{1}{1+{e}^{-{x}_{ij}}}$ was used as the activation of the last layer. The *Adam* algorithm (Kingma and Ba 2014) implementing the stochastic gradient descent approach was used to explore the likelihood function and the *binary crossentropy* loss function was used to quantify the quality of the classification applied to the training data set. The Keras implementation is summarised by Fig. 1.

In order to mitigate the imbalance in class counts observed in the training data set, the above, naïve, algorithm was modified by pre-imposing different weights for the correct and the incorrect SNP class while calculating the loss metric. Following the recommendations from the TensorFlow online manual, a weight for the correct SNP class was estimated as $\frac{1}{2}\cdotp \frac{N_{\mathrm{trueSNP}}+{N}_{\mathrm{falseSNP}}}{N_{\mathrm{trueSNP}}}$ and for the incorrect SNP class as $\frac{1}{2}\cdotp \frac{N_{\mathrm{trueSNP}}+{N}_{\mathrm{falseSNP}}}{N_{\mathrm{falseSNP}}}$, where *N* represents the number of SNPs representing the respective class. This algorithm is referred to as WEIGHTED. Another modification of the NAÏVE algorithm was realised within the frame of construction of the training data set. In particular, SNPs for the incorrect class were randomly sampled with replacement from the pool of all incorrect SNPs. This resulted in the larger number of SNPs representing the incorrect SNP class. In the OVERSAMPLED30 algorithm, the number of incorrect SNPs was equal to 30% of the number of correct SNPs; in the OVERSAMPLED60 algorithm, the number of incorrect SNPs was equal to 60% of the number of correct SNPs and in the OVERSAMPLED100 algorithm, both classes were represented by the equal numbers of SNPs.

### Classification quality metrics

The classification approach comprises the following categories:

- True positive (TP) is defined as the situation when an incorrect SNP was classified as incorrect.
- False negative (FN) is defined as the situation when an incorrect SNP was classified as correct.
- True negative (TN) is defined as the situation when a correct SNP was classified as correct.
- False positive (FP) is defined as the situation when a correct SNP was classified as incorrect.

Based on these categories, two summary statistics were used for the quantification of the quality of classifiers. The F1 metric is given by $F1=\frac{2\mathrm{TP}}{2\mathrm{TP}+\mathrm{FN}+\mathrm{FP}}$, and the SUMSS metric given by $S\mathrm{UMSS}=\frac{\mathrm{TN}}{\mathrm{TN}+\mathrm{FP}}+\frac{\mathrm{TP}}{\mathrm{TP}+\mathrm{FN}}$.

### The estimation of probability cutoff

For each analysed SNP, the original output from the last layer is a probability of a SNP being correct, resulting from the sigmoid activation function. The default true/false class assignment applies the 0.5 probability threshold. For each of the implemented algorithms (NAÏVE, WEIGHTED and the three OVERSAMPLED), in addition to this standard threshold, probability cutoff values were also estimated based on the optimisation of F1 or SUMSS metrics respectively, using the cutpointR package (Thiele and Hirschfeld 2020) implemented in the R. In brief, in this package, separately for each of the five algorithms, a subset of data is sampled with replacement from the original training set of SNPs multiple times. For each such sub-sample, the probability cutoff is represented by the value which yields the highest F1/SUMSS metric. The final estimate is the bootstrap mean of cutoff values from all the bootstrap samples. In order to check the robustness of the cutoff estimates towards the initial data, cutoff estimates were validated by estimating them based on 20 sub-samples of our original training data.

## Results

### Data sets

For the training data set, 2,274,915 SNPs from the three bulls were considered, among which 2,227,995 (97.94%) were correctly identified by the NGS platform (Table 1). Since 24.4% of observations did not have an estimate for the conditional probability of incorrectly called alternative allele (GQ), this metric was not used as an explanatory variable in the DL algorithm. For the validation data set representing the fourth bull, 749,506 correct (98.05%) and 14,940 incorrect SNPs were used (Table 1). A total of 24.2% of observations also did not have an estimate GQ this metric.

**Table 1:** Characteristics of the analysed data sets

SNP	Training data		Validation data	
	Correct	Incorrect	Correct	Incorrect
%	97.94%	2.06%	98.05%	1.95%
Genotype counts				
0/0	882,838	19,725	299,804	6037
0/1	571,549	12,910	193,755	4270
1/1	773,608	14,285	255,947	4633
Mean DP ± SD	37.91 ± 11.66	30.68 ± 13.21	37.61 ± 11.95	33.51 ± 13.08
DP range	1–587	1–457	1–587	1–457
Mean DP2 ± SD	9.53 ± 4.16	6.20 ± 4.09	9.27 ± 3.59	7.13 ± 4.33
DP2 range	1–159	1–113	1–192	1–184
Mean QUAL ± SD	484.15 ± 430.14	365.74 ± 318.29	479.85 ± 429.82	405.4 ± 340.08
QUAL range	10.00–3829.35	10.00–4516.94	10.00–3829.35	10.0–4516.90

### Probabilities of SNP being incorrect, based on training data

**Fig. 2:**
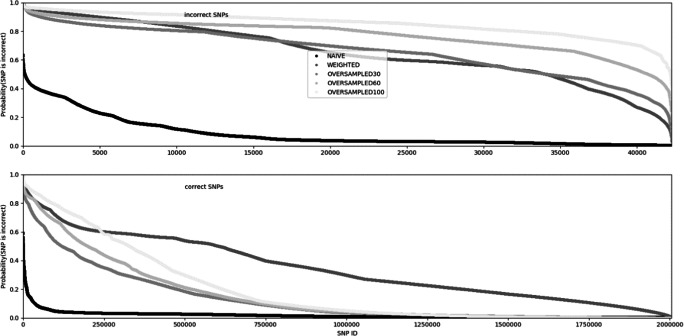
Probabilities of each SNP being incorrect, estimated based on the training data set, by the different algorithms

For each SNP, the probabilities of being incorrectly called, estimated in the training data set by the five models, are depicted in Fig. 2. It is evident that the estimates differ between algorithms, with the two algorithms which mitigate the rare data problem (WEIGHTED, OVERSAMPLED) resulting in generally higher probabilities of a SNP being incorrect. Unfortunately, the examination of the probability curves shows that the NAÏVE algorithm fails to make a distinction between truly correct and incorrect polymorphisms since the empirical probability distributions for each SNP class is quite similar. A visually best differentiation results from the implementation of OVERSAMPLED algorithms, with performance increasing with the balancing of the SNP class counts in the training data set—i.e. the most visually distinct distributions are provided by the OVERSAMPLED100 version.

**Fig. 3:**
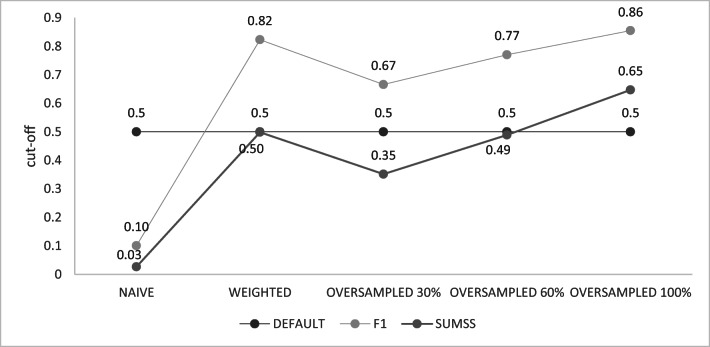
Probability cutoff values for SNP classification into the correct or incorrect group, estimated by the different algorithms based on the optimisation either for the F1 or for SUMSS metric

Note, that in Keras, for a binary (i.e. correct/incorrect) classification, a default cutoff point is 0.5; however, for almost all the applied algorithms, the optimal cutoff points were estimated based on the F1 or SUMSS metrics deviated from this default value (Fig. 3). The most difference was observed when cutoff points were optimised based on the F1 metric and varied between as low as 0.1005 for the NAÏVE algorithm and 0.8550 for the OVERSAMPLED100 algorithm. Cutoff points estimated based on the F1 metric were always higher than those estimated based on SUMSS. However, regardless of which metric was used for the cutoff optimisation, the NAÏVE algorithm resulted in the lowest cutoffs and the highest cutoff values corresponded to the OVERSAMPLED100 algorithm. The accuracy of the cutoff points was examined by re-estimating them based on the bootstrapped sub-samples from the training data set and was very high, as expressed by the standard deviations varying between < 0.001 (NAÏVE for SUMSS) and 0.016 (WEIGHTED for SUMSS).

### Model optimisation

To avoid overfitting, each model was appropriately optimised. For our imbalanced data sets, not only a loss metric, but also precision $\left(\frac{\mathrm{TP}}{\mathrm{TP}+\mathrm{FP}}\right)$ and recall $\left(\frac{\mathrm{TP}}{\mathrm{TP}+\mathrm{FN}}\right)$ metrics were used to choose the optimal number of epochs. Network optimisation was applied based on a subset constructed from 10% of SNPs, stratified (i.e. the same ratio of correct to incorrect SNPs as in the training data set) randomly sampled from the training data set (OPTset). Early stopping parameters were set to control a loss metric of the OPTset, where after 25 epochs without loss improvement, the learning process was stopped. The Supplementary Fig. 1A shows the learning process along with each epoch for the NAÏVE algorithm. It is evident, that this baseline model did not handle our imbalanced data well. According to the loss metric, the network began to overfit after seven epochs. The NAÏVE approach brings high precision at the cost of poor recall close to zero. The WEIGHTED algorithm achieved a much higher recall with a constant level of precision. Based on loss and recall metrics, the WEIGHTED algorithm was trimmed to 30 epochs (Supplementary Fig. 2A). In relation to OVERSAMPLED30 (Supplementary Fig. 3A), OVERSAMPLED60 (Supplementary Fig. 4A) and OVERSAMPLED100 (Supplementary Fig. 5A) algorithms, learning was stopped after 70, 120 and 113 epochs respectively. However, with regard to the OVERSAMPLED algorithms, it is crucial to note that the distributions of metrics will be different because of differences in SNP class percentages between the OPTset and the training data set.

### Classification of training data

**Fig. 4:**
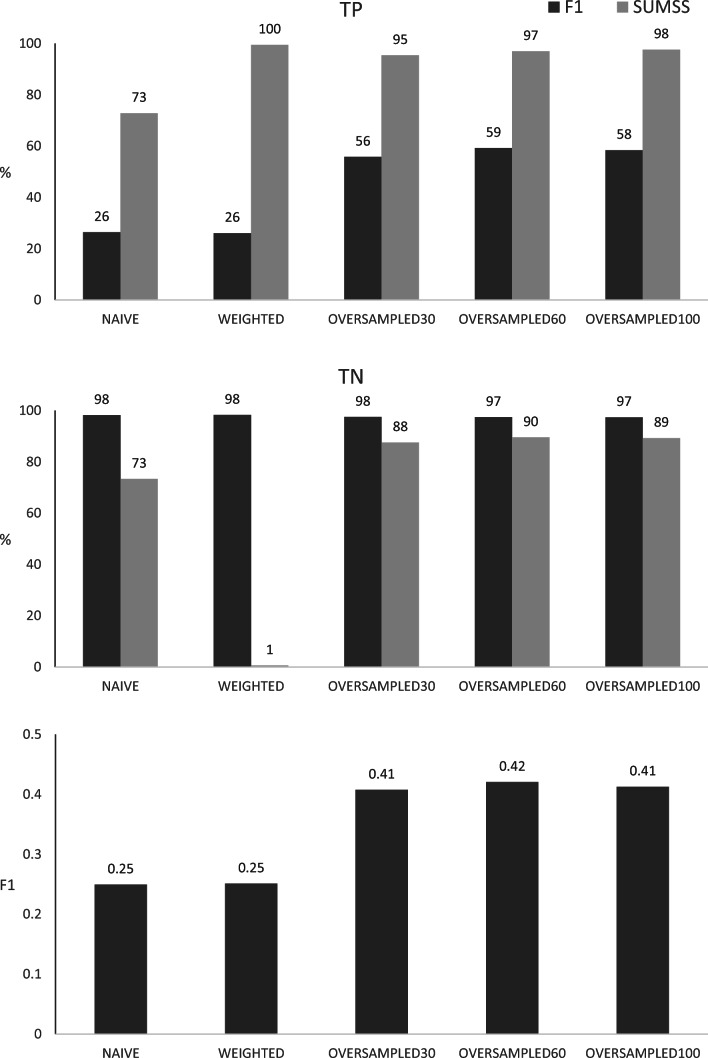
Classification of training data by the different algorithms, based on the probability cutoff thresholds estimated for the F1 or SUMSS metrics. The numbers above columns represent TP—percentages of true positive results, TN—percentages of true negative results, F1—values of the F1 metric

Figure 4 visualises the classification obtained for training data with the final parameters of the classification algorithms. Regardless of the applied probability cutoff (i.e. estimated base on the F1 or on the SUMSS metrics), both—the NAÏVE and especially the WEIGHTED algorithms—do not provide a reasonable classification. Therefore, the approach based on oversampling of the incorrect SNP category emerged to be a better option. No marked differences were observed among the three oversampling schemes tested, but nominally, the best F1 metric of 0.42 was obtained for the most balanced scheme attributed to the OVERSAMPLED60 algorithm. Depending on the probability cutoff applied, this algorithm properly classifies 59.22% of incorrect SNPs and 97.42% of correct SNPs (for the F1 based cutoff estimated to 0.77) or 96.96% of incorrect SNPs and 89.56% of correct SNPs (for the SUMMS based cutoff estimated to 0.49). The latter shall be regarded as the best of the compared classification schemes. Since the probability cutoffs estimated based on the F1 metric were always higher than those based on the SUMMS metric classification based on the former always *favoured*, the proper assignment of correct SNPs while the classification trend based on SUMMS was the opposite.

### Classification of validation data

**Fig. 5:**
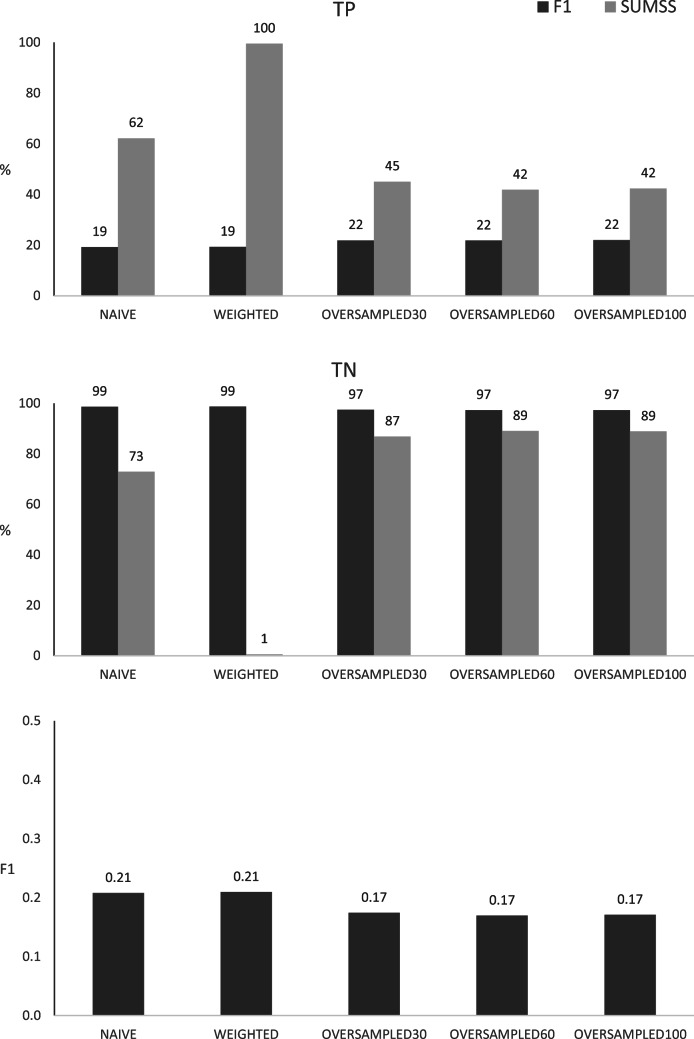
Classification of validation data by the different algorithms, based on the probability cutoff thresholds estimated for the F1 or SUMSS metrics. The numbers above columns represent TP—percentages of true positive results, TN—percentages of true negative results, F1—values of the F1 metric

Obviously, the classification of validation data, depicted in Fig. 5, was not as good as in the training conditions. The F1 metrics dropped down to 0.21 for NAÏVE and WEIGHTED algorithms as well as 0.17 for all OVERSAMPLED algorithms, which was a decrease by 0.04 (NAÏVE, WEIGHTED), 0.24 (OVERSAMPLED30, OVERSAMPLED100) and 0.25 (OVERSAMPLED60). With that, it became evident that the good performance of the OVERSAMPLED algorithms observed on training data was not robust, especially for the detection of incorrect SNPs. In particular, for training data and using the probability cutoff estimated (using the training data set) based on F1, only some 22% of such SNPs were detected by the OVERSAMPLED algorithms. This was a decrease of 33.87% for OVERSAMPLED30, by 36.39% for OVERSAMPLED100 and 37.40% for OVERDSAMPLED60 and thus much higher than the decrease of 7.13% and 6.69% observed respectively for NAÏVE and WEIGHTED algorithms. On the other hand, the proper detection of correct SNPs was on the same level for both analysed data sets (i.e. training and validation). A very similar relation between test-based and validation-based classifications was observed when using probability cutoff values estimated based on the SUMSS metric, visualised in Fig. 5. The F1 metric quantifying the overall performance of the algorithms favoured the basic models—NAÏVE and WEIGHTED, which yielded F1 of 0.21. All three algorithms trained based on OVERSAMPLING of the incorrect SNPs resulted in a lower F1 of 0.17.

## Discussion and conclusions

The classification of rare event data has been a long recognised problem in statistics. Before the era of machine learning, such data was typically attacked either by modification of input data through applying continuity corrections (Sweeting et al. 2004) and re-sampling (Frühwirth-Schnatter and Wagner 2008) or by modification of the underlying logistic regression model by estimates correction and applying different weighting for input data classes (King and Zeng 2001a). Unfortunately, none of those statistical-based approaches resulted in satisfactory handling of the rare event class. A modern extension of handling the problem is to apply the machine learning approach, which with its flexibility towards data structures poses a promising alternative.

NGS-based classification of variants based on the standard output from VCF files is not a new idea. Already in 2013, Durtschi et al. proposed a metric based on the likelihood of three possible genotypes and read depth at a polymorphic site to classify each SNP into four categories expressing different probabilities of being a correct call. Other methods, reviewed by Heydari et al. (2017), aiming not only to identify ambiguous SNP calls but also to correct the original output from variant calling pipelines based on Illumina sequencing, were either based on analysing sequence k-mers or on multiple alignments. In the context of machine learning for SNP classification based on the standard VCF output, Shringarpure et al. (2017) constructed a classifier based on random forests. Deep learning was applied by Ravasio et al. (2018) who estimated the SNP correctness probability and provided a SNP classification tool GARFIELD based on a multi-layer network implemented through the H2O platform (www.h2o.ai). The authors achieved generally high areas under the ROC, varying between 0.63 and 0.98, depending on the analysed platform and coverage. In their unpublished report deposited in the bioRxiv preprint server (2019), Singh and Bhatia applied a series of dense layers to classify data originated from the IonTorrent technology, obtaining a high F1 score of 0.94. The authors circumvented the rare nature of incorrect SNP calls by evaluating only subsets of correct and incorrect variants of equal size.

The experience gained from our analysis of the data is that for a rare event classification problem, like incorrect SNP detection in NGS data, a more parsimonious network, which is less adapted to the specificity of a training data set, is a better, i.e. more robust, option.

## Electronic supplementary material

ESM 1 (DOCX 453 kb)
