# Supplementary material for: The application of deep learning for the classification of correct and incorrect SNP genotypes from whole-genome DNA sequencing pipelines
Source: J Appl Genet. 2020 Sep 29;61(4):607–16. doi: 10.1007/s13353-020-00586-0 (PMC7652806; doi:10.1007/s13353-020-00586-0)
Supplement: Supplementary file 1 — (DOCX 453 kb) [file 13353_2020_586_MOESM1_ESM.docx]

The application of deep learning for the classification of correct and incorrect SNP genotypes from whole genome DNA sequencing pipelines

Krzysztof Kotlarz^1^, Magda Mielczarek^1,2^, Tomasz Suchocki^1,2^, Bartosz Czech^1^, Bernt Guldbrandtsen^3^, Joanna Szyda^1,2^

**Figures in the Supplementary material**

Each figure contains the following metrics:

Loss: the classification loss score.

AUC: area under the receiver operating characteristic curve.

Precision: $\frac{TP}{TP+FP}$

Recall: $\frac{TP}{TP+FN}$

Figure 1A. Optimisation of the NAÏVE algorithm. Train – the training data set, Val – the validation data set.

Figure 2A. Optimisation of the WEIGHTED algorithm. Train – the training data set, Val – the validation data set.

Figure 3A. Optimisation of the OVERSAMPLED30 algorithm. Train – the training data set, Val – the validation data set.

Figure 4A. Optimisation of the OVERSAMPLED60 algorithm. Train – the training data set, Val – the validation data set.

Figure 5A. Optimisation of the OVERSAMPLED100 algorithm. Train – the training data set, Val – the validation data set.

**Figure 1A**

**
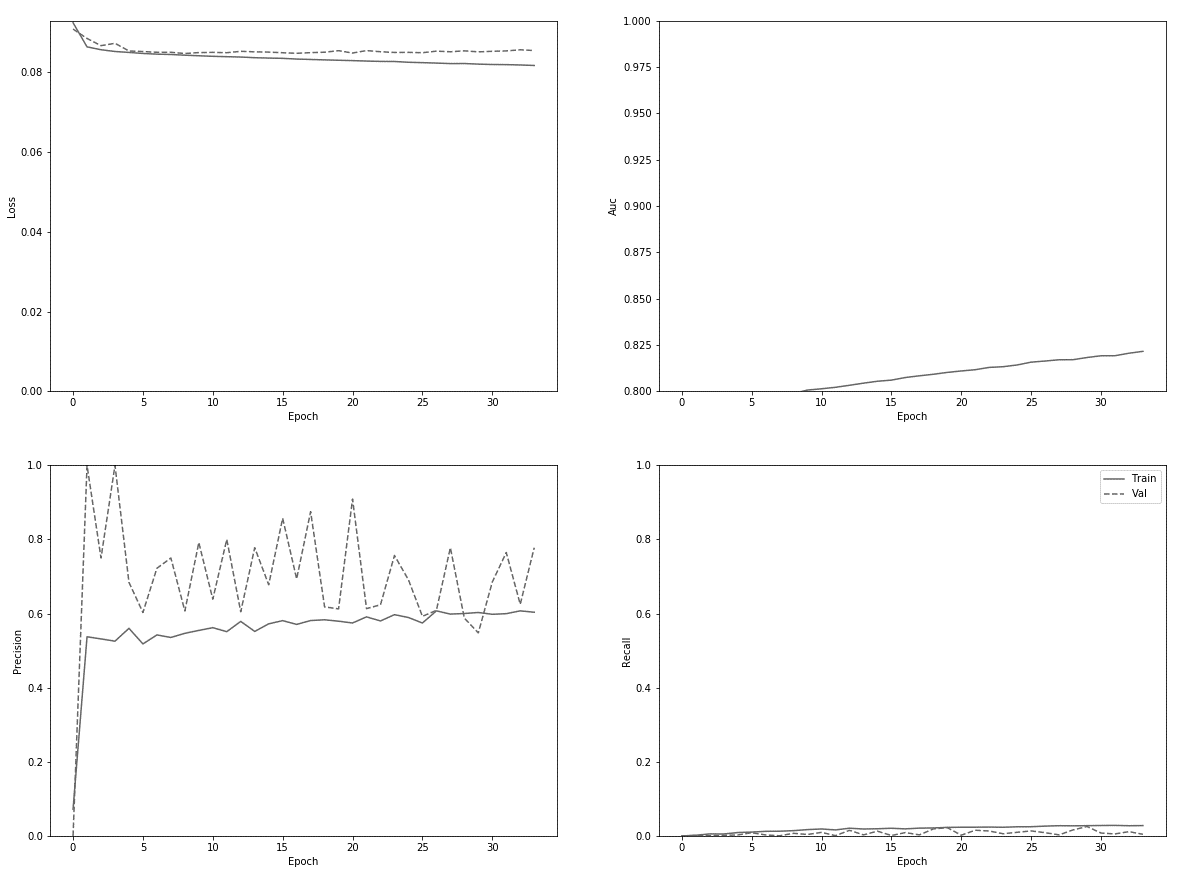
**

**Figure 2A**

**
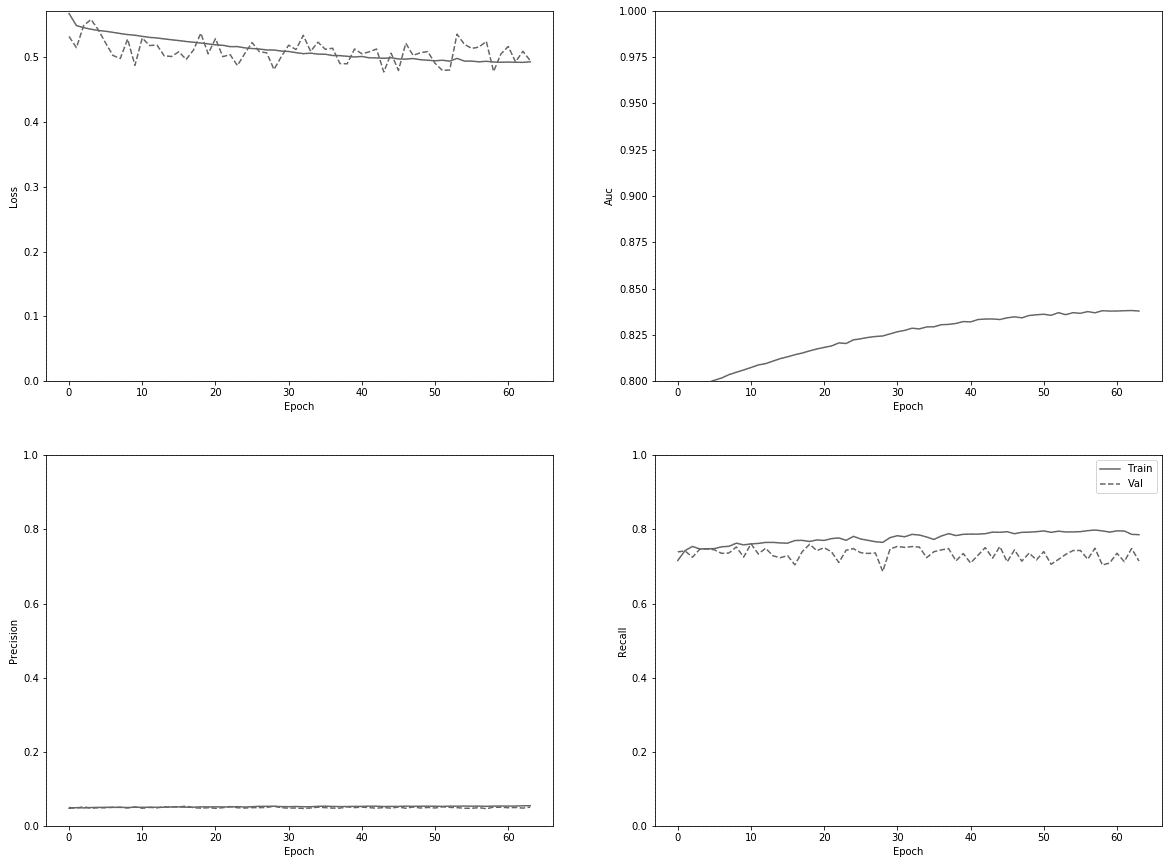
**

**Figure 3A**

**
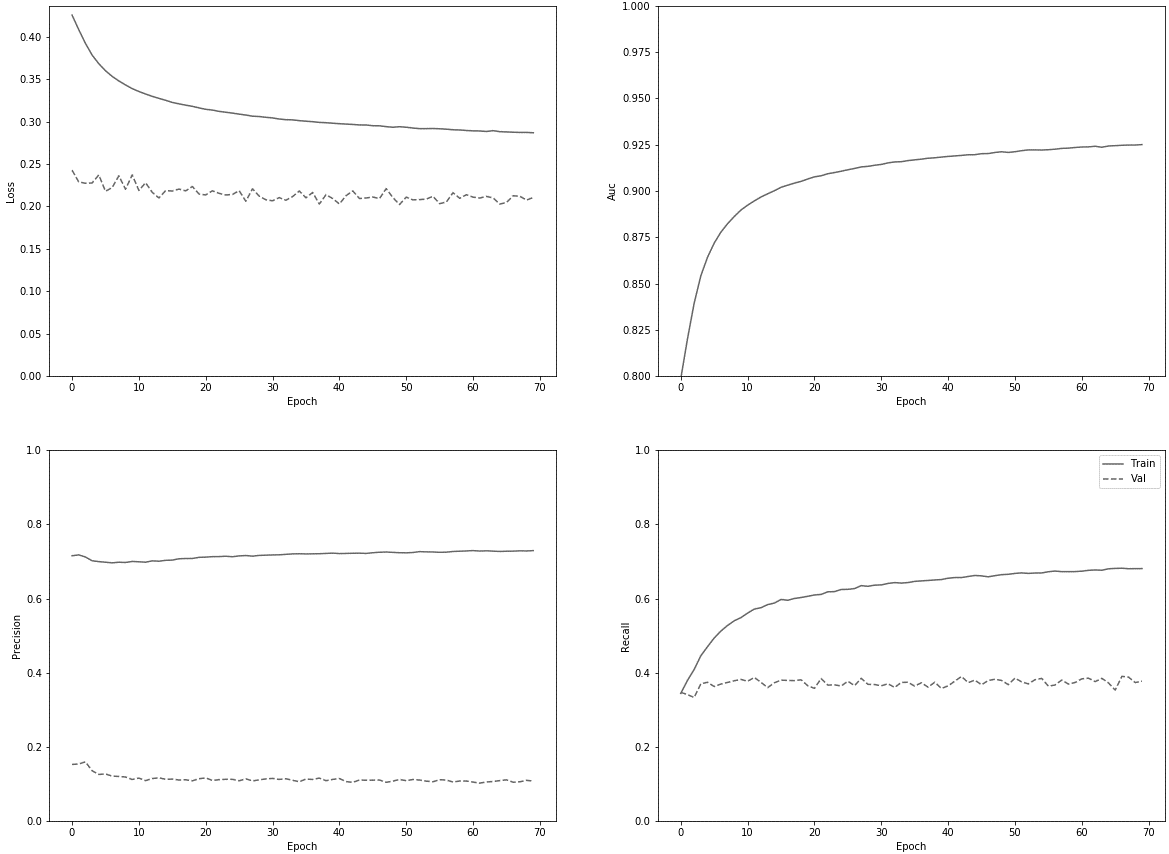
**

**Figure 5A**

**
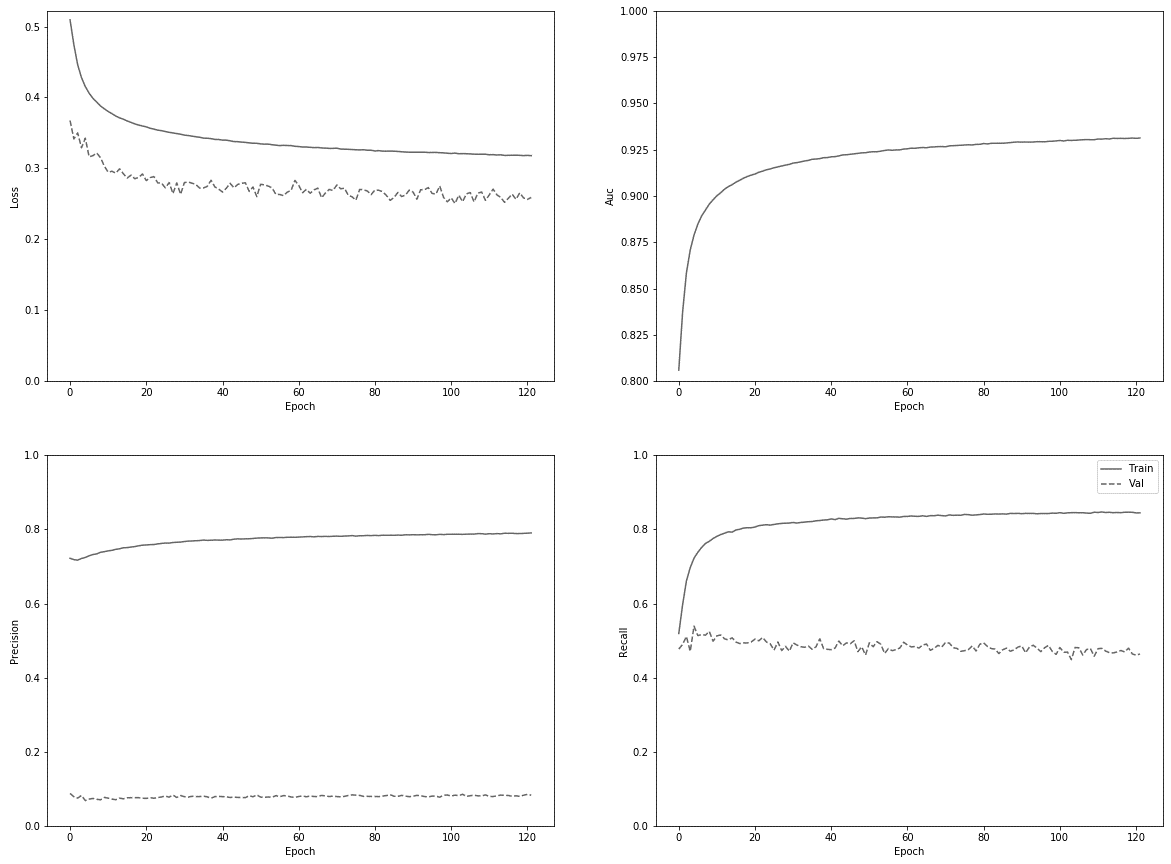
**

**Figure 6A**

**
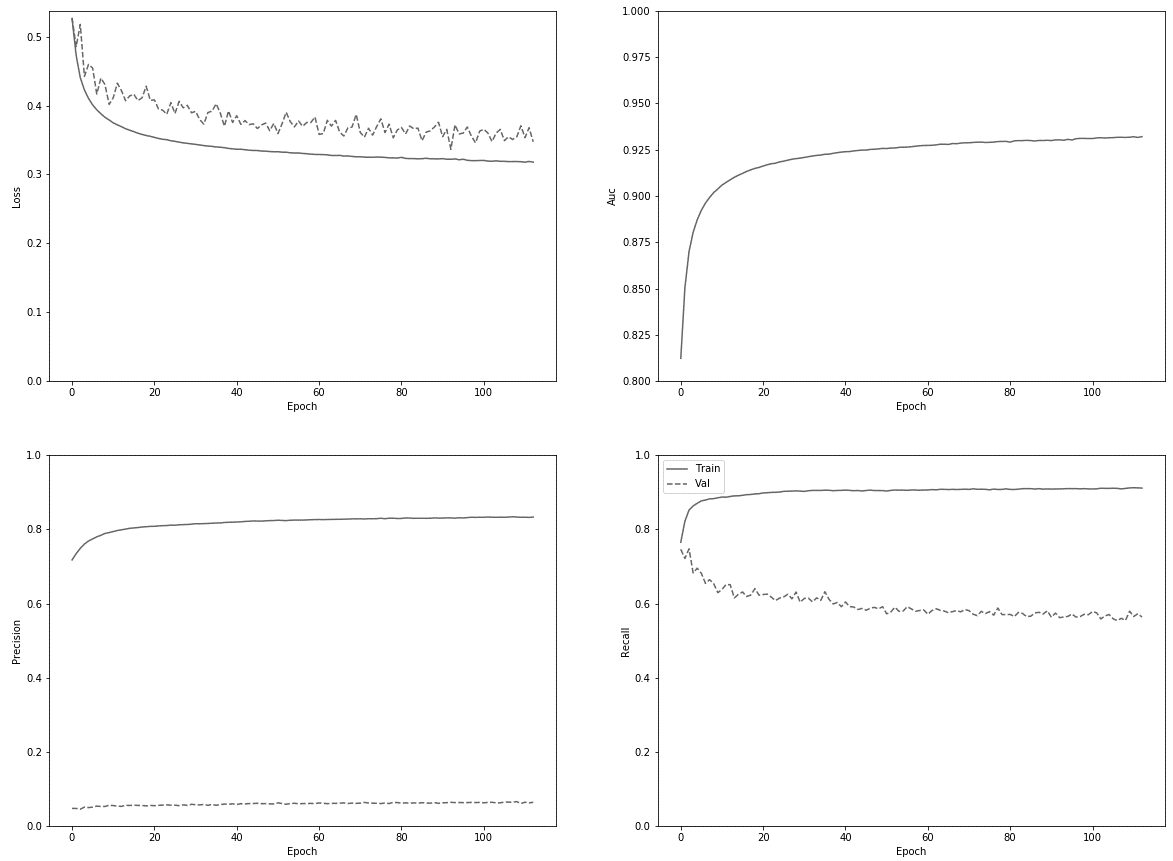
**
